# CINPER: An Interactive Web System for Pathway Prediction for Prokaryotes

**DOI:** 10.1371/journal.pone.0051252

**Published:** 2012-12-07

**Authors:** Xizeng Mao, Xin Chen, Yu Zhang, Spencer Pangle, Ying Xu

**Affiliations:** 1 Computational Systems Biology Lab, Department of Biochemistry and Molecular Biology and Institute of Bioinformatics, University of Georgia, Athens, Georgia, United States of America; 2 BESC BioEnergy Science Center, University of Georgia, Athens, Georgia, United States of America; 3 College of Computer Science and Technology, Jilin University, Jilin, Changchun, China; 4 Key Laboratory of Symbolic Computation and Knowledge Engineering of the Ministry of Education, Jilin University, Jilin, Changchun, China; The Centre for Research and Technology, Greece

## Abstract

We present a web-based network-construction system, CINPER (CSBL INteractive Pathway BuildER), to assist a user to build a user-specified gene network for a prokaryotic organism in an intuitive manner. CINPER builds a network model based on different types of information provided by the user and stored in the system. CINPER’s prediction process has four steps: (i) collection of template networks based on (partially) known pathways of related organism(s) from the SEED or BioCyc database and the published literature; (ii) construction of an initial network model based on the template networks using the P-Map program; (iii) expansion of the initial model, based on the association information derived from operons, protein-protein interactions, co-expression modules and phylogenetic profiles; and (iv) computational validation of the predicted models based on gene expression data. To facilitate easy applications, CINPER provides an interactive visualization environment for a user to enter, search and edit relevant data and for the system to display (partial) results and prompt for additional data. Evaluation of CINPER on 17 well-studied pathways in the MetaCyc database shows that the program achieves an average recall rate of 76% and an average precision rate of 90% on the initial models; and a higher average recall rate at 87% and an average precision rate at 28% on the final models. The reduced precision rate in the final models *versus* the initial models reflects the reality that the final models have large numbers of novel genes that have no experimental evidences and hence are not yet collected in the MetaCyc database. To demonstrate the usefulness of this server, we have predicted an iron homeostasis gene network of *Synechocystis* sp. PCC6803 using the server. The predicted models along with the server can be accessed at http://csbl.bmb.uga.edu/cinper/.

## Introduction

The availability of large-scale *omic* data has allowed accurate elucidation of biological pathways for different prokaryotic organisms in a systematic manner, which has led to the development of a number of pathway databases [1] such as KEGG [2], [3], BioCyc [4] and SEED [5]. These databases provide highly useful information for prediction of biological pathways for prokaryotic organisms in general as the same or similar biological processes across related organisms are typically executed through homologous pathways. These well-elucidated pathways of model organisms could be used to predict at least part of the homologous pathways in other prokaryotic organisms [6]–[8]. A number of such software tools have been developed for pathway prediction through homologous pathway mapping, such as PathwayTools [6], KAAS [7] and RAST [8]. However the usage of these tools has been somewhat limited because such tools may not necessarily give rise to directly useable pathway models since the mapped pathways are often fragmented due to two main reasons: (a) some genes in the selected template pathways could not be mapped to any gene in the target genome; and (b) the target pathways may have components that the template pathways do not have. Such missing pieces in a mapped pathway are often referred to as *pathway holes* [9], [10]. Filling such holes and wiring relevant pathways could be quite challenging as this process not only requires expert knowledge of a specific network but also needs substantial analyses of the available data to rule out models inconsistent with the data [11], [12], and to rank/select genes [13]–[15]. In addition, a user may view the template pathway models in these databases as incomplete and hence decide to reconstruct a model based on his/her own knowledge. Hence a process for pathway reconstruction may take a trained biologist a substantial amount of time and effort to do in an iterative fashion.

We have previously developed a computational tool P-MAP [12] for mapping a biological pathway/network from one prokaryotic organism to another. The program uses both sequence information and predicted operons for the target genome to map a homologous pathway. Using P-MAP, we have made a number of pathway predictions [16]–[19] for *Synechococcus* WH8102. Like other similar programs, P-MAP also faces the same pathway-hole problem as outlined above. In addition, the template pathways may represent only the core part of the target pathway so some genes of the target pathway may not be covered by its homologous pathways in other organisms. Hence, utilization of additional information such as operons, protein-protein interactions and gene expression data represents an important task in pathway prediction. Clearly if gene expression data is available, it can be used to possibly validate a predicted pathway. All these will need to be carried out in order to build a reliable and useful pathway model. To the best of our knowledge, no computer program is currently available in the public domain, which integrates all these data sources and capabilities into one software package to assist a user to construct and validate a biological pathway/network.

We present here an interactive web platform, CINPER, for prediction of a user-specified pathway in a prokaryote, which integrates multiple types of information, including (i) homologous template pathways of multiple related organisms that are in public pathway databases or published papers, (ii) functional annotation of genes, (iii) predicted operons, (iv) protein-protein interactions, (v) phylogenetic profiles and (vi) gene-expression data if available, for the target organism. CINPER essentially automates a manual prediction process [16]–[19] of a biological network by a human expert using the aforementioned tools and data; and does so in an iterative fashion, which involves interactive user inputs, computational prediction, data analyses and result display. During the prediction process, CINPER displays partial prediction results through its graphical user-interface, and prompts the user to enter or search for additional information, and revise a predicted model manually in an interactive manner. If gene-expression data is available, CINPER can assess the consistency between a predicted model and the gene-expression data, and then suggest to the user to enter additional information for revising the inconsistent parts based on the available data. CINPER provides a suite of utility tools supporting automated information retrieval from public databases such as RefSeq [20], KEGG [2], [3], BioCyc [21], SEED [5], DOOR [22] and STRING [23] for finding functional and interaction information of specified genes. The CINPER server can be accessed at http://csbl.bmb.uga.edu/cinper/. Throughout this paper, we will use “pathway” and “networks” interchangeably, with the same meaning.

## Materials and Methods

### Data

All the genome sequences and annotations used here were retrieved from the NCBI FTP server (ftp://ftp.ncbi.nih.gov/genomes/Bacteria, 01-24-2009). The DOOR database [22] was downloaded from the website (http://csbl1.bmb.uga.edu/OperonDB_10142009, v2). The SEED [5] database was downloaded from the SEED FTP server (ftp://ftp.theseed.org/genomes/SEED/, 10-10-2010). The STRING database [23] was downloaded from the STRING website (http://string-db.org/, v8.3). All the data will be updated annually or when a new release is available.

### Software Workflow and Implementation

**Figure 1:**
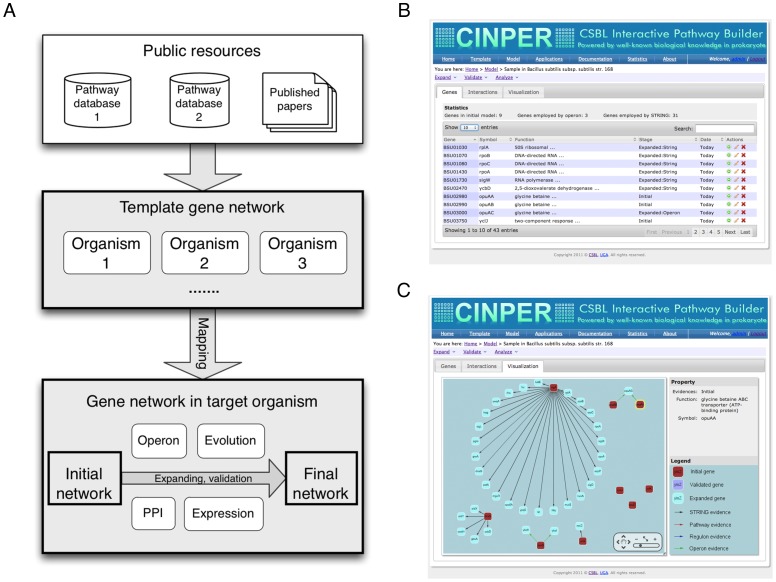
Web interface and workflow of CINPER. (A) a schematic diagram of workflow of CINPER, showing the four-step process of gene-network reconstruction: (1) collection of template networks, (2) construction of initial target network, (3) expansion of initial target network, and (4) validation of the prediction network; (B) a list of all the genes or interactions (not shown) in the target pathway in a sortable and editable table, and (C) a display of the current pathway model shown in a clickable graphics viewer, where each red rectangle represents a gene in the initial pathway model created through orthologous gene mapping from a template pathway; each cyan rectangle represents a gene predicted to be involved in the target pathway based on functional relations with genes in the initial pathway model; and the color of an edge represents the type of a functional relation between two involved genes.

The workflow of CINPER is a four-step process of gene-network construction, which has been well tested and validated in our previous papers [16]–[19]. The process includes: (a) collection of template networks of multiple related organisms from public pathway databases or published literature; (b) construction of initial target network through mapping of template networks using P-Map; (c) expansion of the initial network using multiple types of association information derived from operons, protein-protein interactions and phylogenetic profiles; and (d) validation of the predicted network based on gene expression data, as shown in Figure 1A. The system provides the option for human inputs in an iterative manner in the advanced operating mode mainly to provide an opportunity for the user to decide which computed information should be used for the follow-up calculation, to enter additional information, and to decide on which tools to use to do a specific computation. This allows the user to utilize his/her domain expertise in building a high-quality network model.

CINPER is designed to help to minimize the manual efforts by the user in pathway model construction, as shown in Figure 1B and 1C. The system provides (a) separate web pages in support of construction of template and target networks using dropdown menus; (b) a graphic network display and an interactive webpage to facilitate the user to examine partial results by the automated procedures and provide additional inputs; (c) an interactive web form to facilitate the construction of the initial network from template networks by allowing the user to adjust calculation parameters as well as invoking relevant programs; (d) a webpage with dropdown menus to facilitate user-directed network analyses and calculation, such as making network expansion and computational validation; (e) access to the management system and the online storage system to allow the user to examine the status of their computing jobs and store the results; and (f) a set of web-based utility tools to facilitate visualization using the Cytoscape Web [24] and downstream analyses of the computed models.

CINPER is implemented using the Python programming language (http://www.python.org) and the Django web framework (http://www.djangoproject.com). The advanced interactive features including name completion, dropdown menus, keyword filtering and multiple-column sorting in HTML tables are developed using JavaScript and the jQuery library (http://jquery.com). All the template information and the user account information are stored in a PostgreSQL (http://www.postgresql.org) relational database. CINPER runs on a Linux box (8 Intel Xeon 2.80 GHz and 8 GB RAM), and can also run on Microsoft Windows and Mac OS X. The web site includes a step-by-step tutorial (with screenshots) for general users. Also, to help new users to get started quickly, CINPER provides a sample template and network for the users to try. When a user has no template yet, a clickable hyper link to sample template networks will pop up, and the user can click on the link to make a copy of the sample template network in their work space and try the system with all functionalities that CINPER provides.

## Results and Discussion

### One-click Mode and Advanced Mode

CINPER supports two application modes for different purposes: the *one-click mode* for automated pathway prediction without human intervention, and the *advanced mode*, which involves applications of multiple tools and interactions between the system and the user. In the one-click mode, a user just enters a description about the target process for a selected organism using a set of keywords; and CINPER will predict a pathway model by automatically carrying out the following steps: (i) retrieve relevant (possibly partial) template pathways by searching the entered keywords against the BioCyc [21] and SEED [5] databases; (ii) map the retrieved template pathways to the target genome to give the initial network prediction; and (iii) expand the initial network to fill pathway holes and to recruit additional genes based on operon information retrieved from the DOOR [22] operon database and protein-protein interaction, co-evolution and co-expression information from the STRING [23] functional network database. In the advanced mode, a user can predict a pathway through an interactive manner to control each construction step, each of which is described in the following sections.

### Template Pathway Collection from Multiple Resources

Template collection is the first step in pathway prediction. In this step, a user can import relevant (homologous) pathways from the specified public pathway databases, or enter the relevant genes that have been experimentally determined to be in the target pathway into the pathway-template table through the dropdown menu: “Add genes” on the template-pathway page. To do this, the user can search BioCyc [21] and SEED [5] through CINPER by providing the organism names and keywords related to the target pathway and/or genes, and then select the relevant part of the search results and import them into the pathway-template table, which consists of the possibly involved gene names and functions. To help the user check the details of the search result such as gene functions, CINPER provides hyperlinks to the websites of the relevant pathway databases for each identified gene. In addition, the user can enter information about individual genes one by one or by uploading a list of genes known to be relevant to the target pathway using the GI numbers and PubMed IDs. CINPER provides a utility tool for extracting gene annotation and reference information from the NCBI Genome and PubMed databases and for adding the interactions for any gene pair in the template if they are functionally linked in the pathway models of KEGG [2], [3] or BioCyc [21]. To facilitate fast interactions between the user and the system, CINPER provides a capability for completing an organism’s name based on the first few letters entered by the user.

All identified genes and interactions from the template pathways are listed in a table of the current session, which contains, for each gene and interaction, the gene symbol, its functional description as extracted from the NCBI Genome Database, and supporting evidence that the gene is relevant to the target pathway. Genes from different organisms are merged into one table, leaving to the user to decide the ones to keep or leave out through the user interface. Specifically, the user can select the relevant genes through specifying keywords in the search box in the display window. CINPER provides a utility tool to allow the table to be sorted according to any specified column. For example, a user may wish to find out the number of orthologous genes from different organisms for a particular gene in the table so she/he can sort the table by gene symbols and quickly find out the number. The user can also remove genes and interactions deemed to be irrelevant by clicking on the cross icon or edit the gene and interaction information by clicking the pencil icon.

### Pathway Prediction using Multiple Templates

#### Pathway mapping

CINPER maps the template pathways onto the target genome through the menu “Derive a new model” in the dropdown menu “Go! Model” in the template-pathway page, which activates the P-MAP program [12] for each involved template organism. P-MAP maps genes in each template pathway onto the target genome in such a way that maximizes the overall sequence similarity among homologous genes under the constraint that the mapped genes are grouped into a few operons. P-MAP uses the BLAST-based bi-directional best hit method [25] to predict orthologous genes between template and the target genomes. CINPER wraps the P-MAP program with an intuitive web interface, allowing a user to set the BLAST E-value cut-off (10^−6^ as the default) for orthologous gene search against each organism if needed using the “Organism-specific mapping” form. For the user-specified target genome, CINPER retrieves the operon information from the DOOR operon database [17]. The mapping result consists of the initial pathway model (generally fragmented), in which all the genes are listed together and left for the user to decide if conflicts occur and how to fix them. Functional relationships among mapped genes are copied from their template pathways. Specifically, if two genes of the same template pathway have a direct functional link, e.g., enzymes involved in consecutive reaction steps, their mapped genes will keep the same relationship. Once the initial model is done, the user will be automatically directed to the web page of the pathway model, which can also be accessed by clicking on the “Model” button in the top navigation bar.

We have assessed P-MAP in terms of the number of genes mapped correctly to the target organism on all the pathways having at least 3 genes in MetaCyc [26] under the condition that the pathways are well characterized in both the target and the template organisms and are experimentally validated (so we can compare our predictions with the well-studied pathways). We used *E. coli* as the template organism and *B. subtilis* as the target, and mapped 17 such pathways using BLAST E-value ≤10^−6^ as the cut-off, as shown in Table 1. The performance is measured using *precision* and *recall* defined as follows:
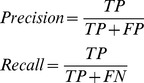
where *TP* is the number of template genes that are mapped to the known target genes, *FP* is the number of template genes that cannot be mapped by P-MAP or that have no matched target genes, and *FN* is the number of missed target genes by P-MAP. For example, for Chorismate biosynthesis I, Precision is equal to 100% by 6/(6+0) (0 is not shown in Table 1), and Recall is equal to 75% by 6/(6+2). Overall P-MAP maps most of the genes with high average *precision* (90%) and *recall* (76%) rates in all 17 pathways correctly, indicating the high quality of the initial models.

**Table 1:** 17 predicted pathways from *E. coli* to *B. subtilis* by CINPER based on MetaCyc.

Pathway	*E.coli*	*B. subtils*	Initial model by P-MAP			Expanding	Final model		
			Total	Precision	Recall	(O|P|X|E)^1^	Total	Precision	Recall
Chorismate biosynthesis I	11	8	6	100%	75%	0|1|0|0	7	27%	87.5%
D-glucuronate degradation	5	4	2	50%	50%	1|0|0|0	3	13%	75%
Galacturonate and glucuronate catabolism	7	6	4	67%	67%	1|0|0|0	5	22%	83%
Peptidoglycan biosynthesis I	11	14	10	100%	71%	0|2|0|0	12	41%	86%
Thiamin biosynthesis I	14	6	6	60%	100%	0|0|0|0	6	16%	100%
Sulfate reduction I	8	8	5	71%	62.5%	3|0|0|0	8	12.5%	100%
Histidine biosynthesis	8	7	6	75%	87%	0|0|0|0	6	33%	87%
1,4-dihydroxy-2-naphthoate biosynthesis I	6	7	5	100%	71%	1|0|0|0	6	75%	87%
Flavin biosynthesis I	6	6	4	100%	67%	1|0|0|0	5	24%	83%
Tryptophan biosynthesis	5	7	5	100%	71%	1|0|0|0	6	50%	87%
NAD biosynthesis I	5	5	5	100%	100%	0|0|0|0	5	26%	100%
5-aminoimidazole Ribonucleotide biosynthesis I	5	6	5	100%	83%	1|0|0|0	6	35.2%	100%
Biotin-carboxyl carrier protein assembly	5	5	5	100%	100%	0|0|0|0	5	15.6%	100%
Pentose phosphate pathway	3	4	3	100%	75%	0|0|0|0	3	37.5%	75%
Heme biosynthesis from uroporphyrinogen-III	4	4	3	100%	75%	1|0|0|0	4	18%	100%
2-ketoglutarate dehydrogenase complex	3	5	3	100%	60%	0|0|0|0	3	13%	60%
2,3-dihydroxybenzoate biosynthesis	3	4	3	100%	75%	0|0|0|0	3	23%	75%
*Average*				90%	76%			28%	87%

The first column is the pathways in MetaCyc with experimental evidences; the second and third columns are the number of genes in *E. coli* and *B. subtilis pathways, respectively*; the fourth to sixth and eighth to tenth columns are the numbers of genes, and precision and recall rates in the initial and final network models, respectively; and the seventh column is the number of genes recruited by different types of functional relationships.

1 O is the number of genes added based on operon information, P is that based on protein-protein interaction (PPI), X is that based on co-evolution phylogenetic profile and E is the number of genes based on co-expression data.

#### Network expansion

The initial model generally represents the core part of the target network; the network expansion step aims to fill the pathway holes and recruit additional genes to make the model more complete. Our strategy is to recruit through “guilt-by-association”, which can be done using the dropdown menu “Expand” in the pathway-model page. CINPER uses four types of information to identify association relationship: co-operon [27], protein interaction, co-evolution [28], and co-expression, each of which can be done by clicking on the corresponding drop-down menu. It is known that an operon typically encodes enzymes catalyzing subsequent reactions in a metabolic pathway or proteins forming a complex [27]. Hence if some genes in an operon are believed to be in a pathway, there is a good chance that the rest of the genes in the operon are also in the same pathway. CINPER has a few built-in databases to provide such association information, namely the DOOR [22] database of predicted operons for 965 bacterial genomes; and the STRING [23] database with large amount of functional relationship information such as protein-protein interactions, co-expression and co-evolution among genes in 534 bacterial genomes. The user can select these two databases in the dropdown menu and recruit genes based on their functional relationships with genes in the initial pathway model. All the new genes will be merged into one list. The user can also upload his/her own association information, presented in a two-column format with every row representing a pair of genes with functional relationship with one gene already in the model and another just recruited, as shown in the clickable sample files in the dropdown menu.

For each pathway model, all the recruited genes are labeled with the source of the association information in the “Source” column in the pathway-model page, and the relevant connections in the graphic network display are color-coded, which allows the user to verify the reliability. If a gene is recruited by different information sources, it is listed in multiple lines accordingly. Notably, the genes recruited by association information may not always be related with the target network [29], and hence validations against the available experimental data are important (see the following section).

We have assessed the usefulness of each information type used for gene recruiting, using the 14 out of the 17 pathway-mapping examples in Table 1 with a less than 100% recall rate. Based on the co-operon information provided by DOOR, protein-protein interaction, co-evolution and co-expression information from the STRING database, 10 of the 14 pathways are correctly expanded, with the average *recall* rate improved from 76% to 87% compared with the initial model. Co-operon information is the most useful while protein-protein interactions and protein-protein interaction seem to be complementary to the operon information based on the number of recruited genes that are verified in MetaCyc. Co-evolution and co-expression data have no contribution possibly due to the nonconserved phylogenetic profiles of the involved genes and the small coverage of conditions used to generate the available gene expression data.

It should be noted that the expanded models have a higher average *recall* rate (87%) but a lower average *precision* (28%) compared the initial models at 76% and 90% respectively. We believe that the reduced precision rate of the expanded model is mostly an artifact resulted from the fact that the pathway models in MetaCyc are far from being complete. For example, we found that the following items are relevant with the target pathways but are not in MetaCyc, such as *HemY* (BSU10140) that is a peripheral membrane protein essential for *protoheme* IX synthesis [30] and may be related with the *heme* biosynthesis from uroporphyrinogen-III pathway in Table 1, *ThiF* (BSU11700) that is a thiamine biosynthesis protein [31] and may be related with the thiamin biosynthesis I pathway, and *HisH* (BSU34890), an imidazole glycerol phosphate synthase and *HisZ* (BSU34930) a histidyl-tRNA synthetase, both of which are involved in the histidine biosynthesis [32] and may be related with the histidine biosynthesis pathway, suggesting that CINPER could effectively recruit additional relevant genes into the target pathways, for which the additional experimental validation is needed.

#### Network validation

CINPER allows a user to assess the consistency between a predicted network and the available gene expression data using the dropdown menu “Validate” in the network pathway-model page. From the menu, the user can select and upload a one-column file consisting of up- or down-regulated genes under conditions known to trigger changes in expressions of genes of the target pathway, which are identified in advance by the user from some relevant microarray data. If a gene in the expanded network model is included in the uploaded file, this gene is marked as “validated” in the column of “Evidence”, otherwise “predicted”.

### An Application Example to Reconstruction of Iron Homeostasis Network

We have applied CINPER to predict the iron homeostasis network in *Synechocystis* PCC6803. Iron plays an important role in living organisms in general, and it is an integral part of the heme structure and a cofactor in Fe-S proteins, which protect the cell from oxidative and nitrosative stresses and are involved in nitrogen fixation, hydrogen production and consumption, photosynthesis, and methanogenesis [33]. Excessive iron can cause damage to the cell. To avoid this, bacteria maintain homeostasis on intracellular iron using multiple mechanisms such as regulating the expression levels of genes involved in iron uptake, using intracellular iron-storage proteins, selective expression of iron-dependent and iron-independent enzymes during cell growth in iron-replete and iron-deplete environments, respectively [33], and detoxification of iron by specific efflux systems [34].

**Figure 2:**
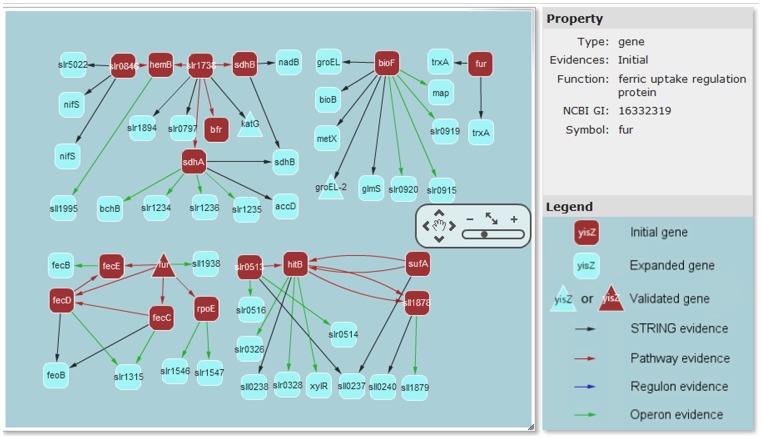
A screenshot of iron homeostasis network in *Synechocystis* PCC6803 predicted by CINPER. The window of network visualization consists of three parts: the left part is a zoomable graphic display, in which a small operation panel in the middle right side can zoom in or out the display; the right upper part is the information for the selected gene in the left graphic display, such as the gene symbol, GI number and gene function; and the right bottom part is a legend to define the meanings of the shapes in the left part: round rectangles are predicted genes, among which red ones are genes in the initial network and the cyan ones are genes recruited through functional relationship; triangles are validated genes using gene expression data; color-coded arrows between two genes represent different interaction types, red for genes in the same pathway, green for genes in the same operon, blue for genes in the same regulon and black for genes with functional relationships as defined by the STRING database, such as protein-protein physical interaction, similar phylogenetic profile or expression profile.

We have collected, through an extensive literature search, 27 template genes known to be involved in the iron response systems in four bacteria: *Sinorhizobium meliloti* (simply *S. meliloti*), *Escherichia coli* K12 (*E. coli*), *Prochlorococcus marinus* MED4 (*P. marinus*) and *Synechocystis* PCC6803 (*S.* PCC6803) (see File S1). 57 iron-related genes are predicted in *S.* PCC6803 using CINPER, of which 17 genes are in the initial network, and 40 genes are recruited based on co-operon information (20 genes) and protein-protein interactions (20 genes), respectively, as shown in Figure 2. It should be noted that we have predicted two Fur-like transcription regulators that have been verified in the published paper [35], and other three pairs of genes with same gene symbols (*nifS*, *sdhB*, *trxA*) but different synonyms. We also validated our prediction using public transcriptomic data under iron limitation conditions [36], [37]; 3 genes (1 is from initial genes, and 2 are from protein-protein interactions) are experimentally verified. The following shows the reconstruction procedure of the iron homeostasis network step-by-step:

1. Create a new blank template by clicking on the “New template” button in the home page of template networks.
2. Collect template networks as listed in File S1:
  - Add the template genes in two ways through the “Add genes” menu: (i) search the SEED and BioCyc databases using keyword “iron”; and (ii) enter manually any identified genes.
  - Add the template interactions through the “Add interactions” menu: (i) search the KEGG pathway database using keyword “iron”; and (ii) enter manually any identified interactions.
  - Revise the template networks by examining the genes and interactions in the corresponding panels and delete irrelevant genes and interactions.
3. Construct the initial network model by clicking on “Derive a new model” in the “Go! Model” menu and adjust the parameter values for each organism if needed. The user will be directed to the web page of the derived initial network model.
4. Expand the initial network by using different association information through the “Expand” menu, including the DOOR operon database, the STRING functional relation database, or user’s own regulon information.
5. Validate the predicted network by uploading and applying the available gene expression data through the “Validate” menu.

**Figure 3:**
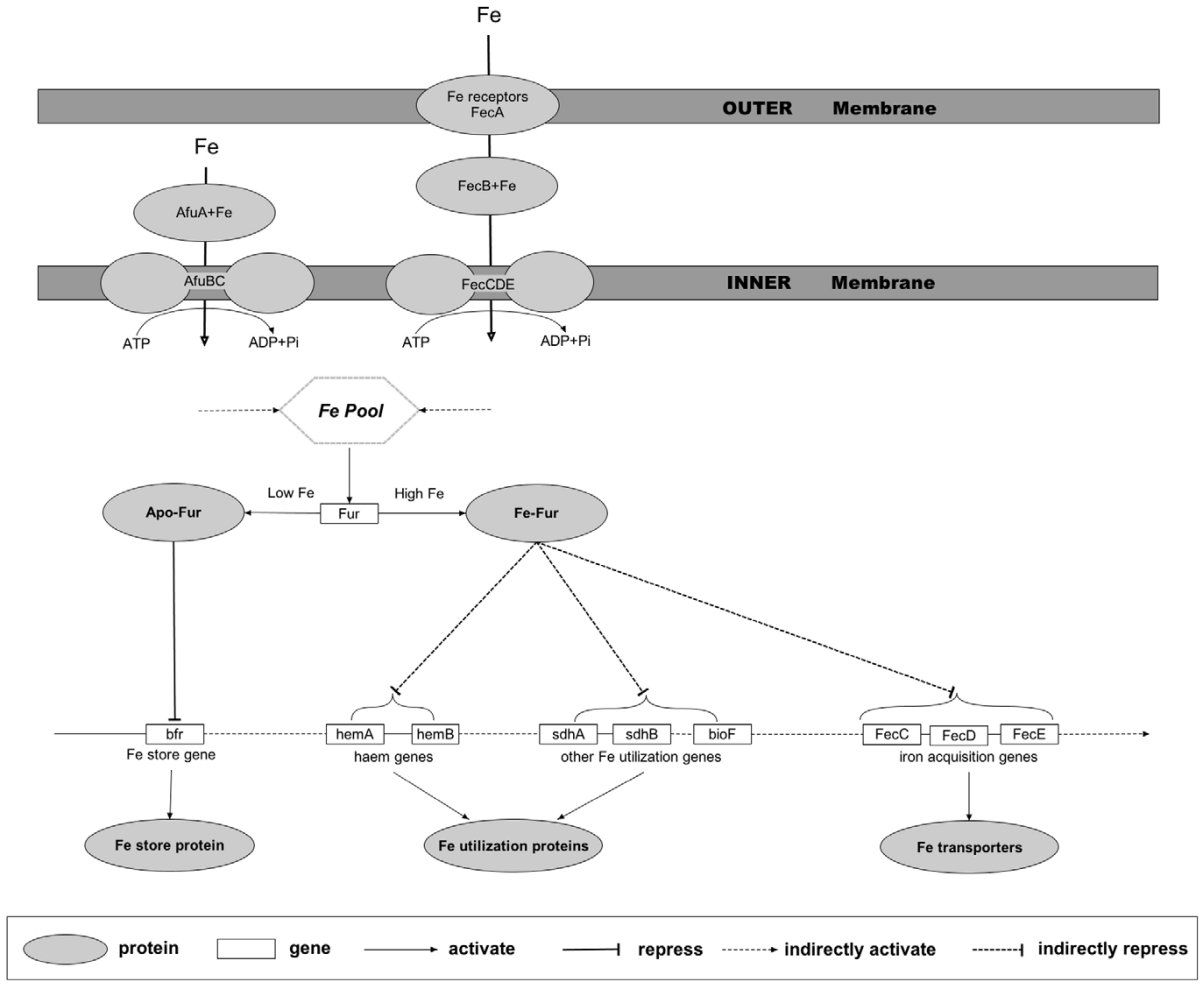
A schematic diagram of iron homeostasis network in *Synechocystis* PCC6803, drawn using the PathVisio program [**40**], showing that iron homeostasis is regulated by the global regulator Fur: when the cell is replete with iron, its bounded form (*Fe-Fur*) represses the transcription of iron transporters; and when the cell is low in iron, its iron-free form (*apo-Fur*) activates iron transporters and repress the transcription of iron storage and utilization proteins to increase the intracellular iron. Proteins are represented as ellipses, and genes are rectangles; arrows are activation processes, and T-bars are repression processes; and dashed lines indicate indirect or hypothetical processes.

The model predicted for iron homeostasis in *S.* PCC6803 by the above procedure consists of the following components (see Figure 3):

1. Global regulator: *Fur* (sll0567 and sll1937) [35] maintains iron homeostasis by sensing changes in intracellular iron concentration and regulating the expression of iron-related genes. When the cell is replete with iron, the bounded form of Fur with iron represses the transcription of the iron transporters; when the cell is low in iron, iron-free form (apo-Fur) activates iron transporters and repress the transcription of the iron storage and utilization proteins to increase the intracellular iron.
2. Iron transporters: *AfuABC* (slr1295, slr0327 and sll1878) [38] and *FecABCDE* (slr1315, slr1319, slr1316, slr1317, slr1318) [39] uptake iron from the periplasm and outside of the outer membrane, respectively, into the cell;
3. Iron storage protein: *bfr* (slr1890);
4. Iron utilization proteins: Heme proteins including *hemAB* (sll1995, sll1994) and other iron utilization proteins, namely *sdhAB* (slr1233 and sll1625) and *bioF* (slr0917).

All the data relevant to this example can be downloaded freely from http://csbl.bmb.uga.edu/~xizeng/research.php?p=cinper1.

## Concluding Remarks

We have developed an interactive web-based platform CINPER for gene network prediction, based on information from homologous pathways from related organisms, as well as specific information about the target pathway and general information about functional associations relevant to known genes in the target pathway. CINPER essentially automates an iterative manual prediction process by a domain expert using a set of tools and experimental data, and does so through an iterative process, which involves data analysis, computational prediction and user inputs. CINPER can be used for multiple purposes, including (i) construction of a desired gene network and (ii) pathway-hole filling. Evaluation of the program on 17 well-studied pathways in the MetaCyc database shows that it achieves an average recall rate at 87%, and an average precision rate at 28%, which the low precision rate, we believe, is mostly due to the fact that the pathway models in MetaCyc are far from being complete.

CINPER is specifically designed to help to minimize the manual work by a user when reconstructing a network model using a set of automated tools and based on available data. We believe that this program provides a useful capability to prokaryotic biologists to build pathway models in a reliable and efficient manner. Further development of the program will be focused on updating data regularly whenever new versions are available and including additional pathway and functional relation information and improving its graphic network displays.

## Supporting Information

File S1 **Template networks of iron homeostasis.**
(DOC)
