# Supplementary material for: CINPER: An Interactive Web System for Pathway Prediction for Prokaryotes
Source: PLoS One. 2012 Dec 7;7(12):e51252. doi: 10.1371/journal.pone.0051252 (PMC3517448; doi:10.1371/journal.pone.0051252)
Supplement: File S1 — Template networks of iron homeostasis. (DOC) [file pone.0051252.s001.doc]

# Template networks for iron homeostasis network

# Background

Iron is an integral part of heme structure and a cofactor in Fe-S proteins, which protects the cell from oxidative and nitrosative stresses (catalases, peroxidases, oxygenases), nitrogen fixation (nitrogenases), hydrogen production and consumption (hydrogenases), photosynthesis, and methanogenesis . Excessive iron can cause damage to the cell, and hence, bacteria have evolved to maintain intracellular iron homeostasis by regulating the expression levels of genes involved in iron uptake, using intracellular storage proteins, selective expression of iron-dependent and iron-independent enzymes in cell growth in iron-replete and iron-deplete systems respectively , and detoxification of iron by specific efflux systems .

Fur (Ferric uptake regulator) is the best-known transcription regulator for maintaining iron-homeostasis in bacteria, which is well studied in γ-proteobacteria, β-proteobacteria, bacilli and cyanobacteria. Fur interacts with ferrous iron under iron-replete conditions, and thus represses the expression of its target genes by binding to the conserved five prime sequences named FUR box . It has been reported that Fur can repress more than 100 genes in iron-replete conditions in *E. coli*: outer membrane iron receptors (*OMP*), ferrous iron transporters (*feoAB*) and ferric iron transporters *fbpABC* and *ftr/chpA*, iron storage (*bfr*), heme synthesis (*hemA*), and iron-utilizing enzymes (*sdh*, *nuo*) .

Irr is a global transcriptional regulator of iron-uptake and metabolism in α-*proteobacteria*, and its sequence motif of five-prime binding region is shared by most iron uptake and storage genes, as well as other genes of iron metabolism, such as *suf* involved in Fe-S synthesis, *hemA* involved in heme biosynthesis, and the operons encoding iron-containing enzymes . In addition, *RirA* is another transcriptional regulator of iron uptake and metabolism in the *Rhizobiales* sub-class of *α-proteobacteria* , in which *RirA* represses the expression of the genes of ferrous iron and heme transport, siderophore biosynthesis and transport, and Fe-S cluster biosynthesis .

# Template networks

We have collected, through literature search, 27 genes known to be involved in the iron response network in four species, *Sinorhizobium meliloti* (*S. meliloti*) , *Escherichia coli* K12 (*E. coli*) , *Prochlorococcus marinus* MED4 (*P. marinus*) , and *Synechocystis sp.* PCC6803 (*S.* PCC6803*)* , for which the iron response network has been well studied. The following summarizes what is known about each of the iron response networks in the 4 species (see Table S1 for details):

- ***E. coli***: has 8 genes in total, encoding a ferric citrate outer membrane porin (*fecA*), a citric-dependent ferric transporter complex (*fecBCDE*), a RNA polymerase, a ferric regulatory protein, and a regulator of *fecBCDE* operon respectively .
- ***P. marinus***: has 4 genes in total, encoding an *ABC*-type ferric iron transporter and one regulator known to be involved in iron uptake systems, iron storage ferritins, Heme synthesis and iron cofactors .
- ***S.* PCC6803**: has 4 genes encoding an *ABC*-type ferric iron transporter .
- ***S. meliloti***: have 11 genes in total, involved in iron uptake systems, iron storage ferritins, Fe-S biogenesis, heme synthesis, and iron cofactors .

Table S1. Components in the template models.

|  | **Synonym** | **GI** | **Symbol** | **Function** | **Pubmed ID** |
| --- | --- | --- | --- | --- | --- |
| *P. marinus* | PMM1164 | 33861720 | *futA* | *ABC*-type ferric iron | 19487728 |
|  | PMM0489 | 33861046 | *futB* | transporter | 19487728 |
|  | PMM0803 | 33861360 | *futC* |  | 19487728 |
|  | PMM0637 | 33861194 | *fur* | Iron uptake repressor, | 19487728 |
|  |  |  |  | maintaining iron homeostasis |  |
| *S. meliloti* | SMc02510 | 15966778 | *fur* | Ferric uptake regulator | 17173478 |
|  | SMc00329 | 15964002 | *irr* | Iron responsive regulator, repressor of the heme biosynthesis gene *hemB* in iron-limited cells | 17173478 |
|  | SMc00785 | 15964483 | *rirA* | Iron responsive regulator, represses expression of genes involved in ferrous and heme transport, siderophore biosynthesis and transport and the synthesis of Fe-S clusters | 17173478 |
|  | SMc03786 | 15966922 | *bfr* | Bacterioferritin, iron storage | 17173478 |
|  | SMc03104 | 15966742 | *hemA* | heme synthesis | 17173478 |
|  | SMc01766 | 15964957 | *hemB* |  | 17173478 |
|  | SMc02465 | 15966823 | *sdhA* | Succinate Dehydrogenase, iron | 17173478 |
|  | SMc02466 | 15966822 | *sdhB* | utilizing enzyme | 17173478 |
|  | SMc02463 | 15966825 | *sdhC* |  | 17173478 |
|  | SMc02464 | 15966824 | *sdhD* |  | 17173478 |
|  | SMc00775 | 15964473 | *fbpB* | Ferric iron transporter | 17173478 |
| *S.* PCC6803 | slr1295 | 16329434 | *futA1* | *ABC*-type ferric iron | 11522907 |
|  | slr0513 | 16331793 | *futA2* | transporter | 11522907 |
|  | slr0327 | 16331231 | *futB* |  | 11522907 |
|  | sll1878 | 16330805 | *futC* |  | 11522907 |
| *E. coli* | b4293 | 16132114 | *fecI* | *RNA* polymerase, σ^19^ factor, directs *fecABCDE* | 19487728 |
|  | b4292 | 16132113 | *fecR* | Regulator of *fecABCDE* operon | 19487728 |
|  | b4291 | 16132112 | *fecA* | ferric citrate outer membrane porin | 19487728 |
|  | b4290 | 162135917 | *fecB* | Citrate-dependent ferric  transporter | 19487728 |
|  | b4289 | 16132110 | *fecC* |  | 19487728 |
|  | b4288 | 16132109 | *fecD* |  | 19487728 |
|  | b4287 | 16132108 | *fecE* |  | 19487728 |
|  | b0683 | 16128659 | *fur* | Repress *FecI* under high iron but not upon iron starvation | 19487728 |

# References

1. Andrews SC, Robinson AK, Rodriguez-Quinones F (2003) Bacterial iron homeostasis. FEMS Microbiol Rev 27: 215-237.

2. Grass G, Otto M, Fricke B, Haney CJ, Rensing C, et al. (2005) FieF (YiiP) from Escherichia coli mediates decreased cellular accumulation of iron and relieves iron stress. Arch Microbiol 183: 9-18.

3. Rodionov DA, Gelfand MS, Todd JD, Curson AR, Johnston AW (2006) Computational reconstruction of iron- and manganese-responsive transcriptional networks in alpha-proteobacteria. PLoS Comput Biol 2: e163.

4. Todd JD, Wexler M, Sawers G, Yeoman KH, Poole PS, et al. (2002) RirA, an iron-responsive regulator in the symbiotic bacterium Rhizobium leguminosarum. Microbiology 148: 4059-4071.

5. Scanlan DJ, Ostrowski M, Mazard S, Dufresne A, Garczarek L, et al. (2009) Ecological genomics of marine picocyanobacteria. Microbiol Mol Biol Rev 73: 249-299.

6. Wagegg W, Braun V (1981) Ferric citrate transport in Escherichia coli requires outer membrane receptor protein fecA. J Bacteriol 145: 156-163.

7. Katoh H, Hagino N, Ogawa T (2001) Iron-binding activity of FutA1 subunit of an ABC-type iron transporter in the cyanobacterium Synechocystis sp. Strain PCC 6803. Plant Cell Physiol 42: 823-827.
